# Inhibition of HIV-1 Infection by Human α-Defensin-5, a Natural Antimicrobial Peptide Expressed in the Genital and Intestinal Mucosae

**DOI:** 10.1371/journal.pone.0045208

**Published:** 2012-09-27

**Authors:** Lucinda Furci, Monica Tolazzi, Francesca Sironi, Lia Vassena, Paolo Lusso

**Affiliations:** 1 Unit of Human Virology, Department of Biological and Technological Research, San Raffaele Scientific Institute, Milan, Italy; 2 Department of Medical Sciences, University of Cagliari School of Medicine, Cagliari, Italy; Imperial College London, United Kingdom

## Abstract

**Background:**

α-defensin-5 (HD5) is a key effector of the innate immune system with broad anti-bacterial and anti-viral activities. Specialized epithelial cells secrete HD5 in the genital and gastrointestinal mucosae, two anatomical sites that are critically involved in HIV-1 transmission and pathogenesis. We previously found that human neutrophil defensins (HNP)-1 and -2 inhibit HIV-1 entry by specific bilateral interaction both with the viral envelope and with its primary cellular receptor, CD4. Despite low amino acid identity, human defensin-5 (HD5) shares with HNPs a high degree of structural homology.

**Methodology/Principal Findings:**

Here, we demonstrate that HD5 inhibits HIV-1 infection of primary CD4^+^ T lymphocytes at low micromolar concentration under serum-free and low-ionic-strength conditions similar to those occurring in mucosal fluids. Blockade of HIV-1 infection was observed with both primary and laboratory-adapted strains and was independent of the viral coreceptor-usage phenotype. Similar to HNPs, HD5 inhibits HIV-1 entry into the target cell by interfering with the reciprocal interaction between the external envelope glycoprotein, gp120, and CD4. At high concentrations, HD5 was also found to downmodulate expression of the CXCR4 coreceptor, but not of CCR5. Consistent with its broad spectrum of activity, antibody competition studies showed that HD5 binds to a region overlapping with the CD4- and coreceptor-binding sites of gp120, but not to the V3 loop region, which contains the major determinants of coreceptor-usage specificity.

**Conclusion/Significance:**

These findings provide new insights into the first line of immune defense against HIV-1 at the mucosal level and open new perspectives for the development of preventive and therapeutic strategies.

## Introduction

With 2.6 million new infections in 2010, two thirds of which (69%) in sub-Saharan Africa, the HIV-1 pandemic remains one of the most important public health challenges worldwide [1]. The limited accessibility to expensive last-generation antiviral drugs and, most of all, the lack of a protective HIV-1 vaccine represent two formidable obstacles for the control of this infection [2]. Since more than 70% of the individuals living with HIV-1 are young women (aged 15–24 years) who acquired the infection through heterosexual contacts [1], effective prophylactic strategies, such as HIV microbicides, could be effective in preventing virus transmission at the mucosal level. The mucosal surface not only poses a physical barrier against pathogens but also hosts diverse defensive mechanisms of natural immunity. Thus, novel vaccination and prevention strategies might benefit from the elucidation of the innate defensive mechanisms that control the early events in HIV-1 invasion at mucosal sites [3].

Studies of vaginal transmission of simian immunodeficiency virus (SIV) demonstrated that between 100- and 1000-fold more virus is required to establish infection in macaques by vaginal application compared to intravenous inoculation [4]. Similar values were obtained from the study of a large cohort of 235 monogamous, HIV-discordant couples in Uganda [5], indicating that the genital mucosal tissue represents in itself a natural obstacle to infection [6]. This circumstantial evidence has been confirmed experimentally by the finding that vaginal fluids inhibit HIV-1 infection in cervicovaginal tissue models *ex vivo* [7]. The majority of such antiviral activity was identified in the cationic polypeptide fraction, which contains several natural antimicrobial proteins and peptides that act as effectors of the innate host defense and display to variable extent anti-HIV activity [8], [9]. These include lysozyme, lactoferrin, secretory leukoprotease inhibitor (SLPI), and defensins [10]. Moreover, cervicovaginal secretions collected from HIV-uninfected Kenyan commercial sex workers, depleted of the IgA fraction, where shown to neutralize primary HIV-1 isolates, and their neutralizing activity was correlated with the levels of human neutrophil peptide (HNP) 1-3 and LL-37 [11], [12].

Human α-defensins are short (29–35 aa.) peptides with a net positive charge and a distinctive 6-cysteine motif that imposes a characteristic β-sheet structure [13]. Human neutrophil peptides (HNP) 1–3 and human defensins (HD) 5 have only modest amino acid sequence identity (40%) but share a common topological structure and similar amphiphilic properties [14]. Alpha-defensins are expressed predominantly by neutrophils (HNP 1–4) and epithelial cells (HD5 and 6). HNP-1 to 3, which in vaginal fluids derive from infiltrating neutrophils, are natural antibiotic peptides that play an important role as a first line of defense against invading pathogens by acting as broad-spectrum antibacterial, antifungal, and antiviral effector molecules [15], as well as by enhancing certain adaptive immune responses [16]. Their antiviral activity extends from enveloped viruses like herpes simplex virus, cytomegalovirus, influenza A virus, vesicular stomatitis virus and HIV-1 [17], to non-enveloped viruses like papillomavirus and adenovirus [18]. These effects are likely mediated by different mechanisms, which are still incompletely understood. Alpha-defensins 1 and 2 (HNP1-2) appear to interfere with HIV-1 replication via different mechanisms, including inhibition of the viral replication cycle and blockade before HIV-1 entry into target cells [17], [19], [20], [21]. We previously reported that inhibition by HNP-1-2 occurs at the early steps of the viral infectious cycle, demonstrating that these defensins directly interfere with the interaction between gp120 and its cellular receptor CD4 [19]. This antiviral mechanism of HNP1 was recently confirmed by Demirkhanyan and colleagues who demonstrated that HNP1 inhibited virtually every step of viral entry: including Env binding to CD4 and coreceptors; refolding of Env into the final 6-helix bundle structure and productive HIV-1 uptake [21]. Besides inhibition of entry, Seidel and colleagues also showed a second mechanism of action occurring several hours after HIV-1 entry at a step downstream of HIV-1 cDNA formation [20].

Originally described only in specialized secretory cells, Paneth cells within intestinal crypts, HD5 was later found in other mucosal tissues, including the female lower genital tract [22]. Here, HD5 is localized in apical secretory granules of the columnar epithelium and is released into the lumen reaching peak concentrations during the early secretory phase of the menstrual cycle [23]. Recently, it has been reported that HD5 has antiviral properties as it specifically inhibits herpes simplex virus, papillomavirus [24], BK virus [25], and adenovirus [26]. In contrast, a surprising enhancing effect on HIV-1 infection was observed with HD5 and HD6 in tests performed *in vitro* in the presence of bovine serum [27], [28]. In this study, we explored the hypothesis that HD5 could act as a natural HIV-1 inhibitor and thereby potentially act as a natural obstacle to HIV-1 transmission in the female lower genital tract.

## Results

### α-defensin-5 Inhibits HIV-1 Replication in Primary CD4^+^ T Lymphocytes

**Figure 1:**
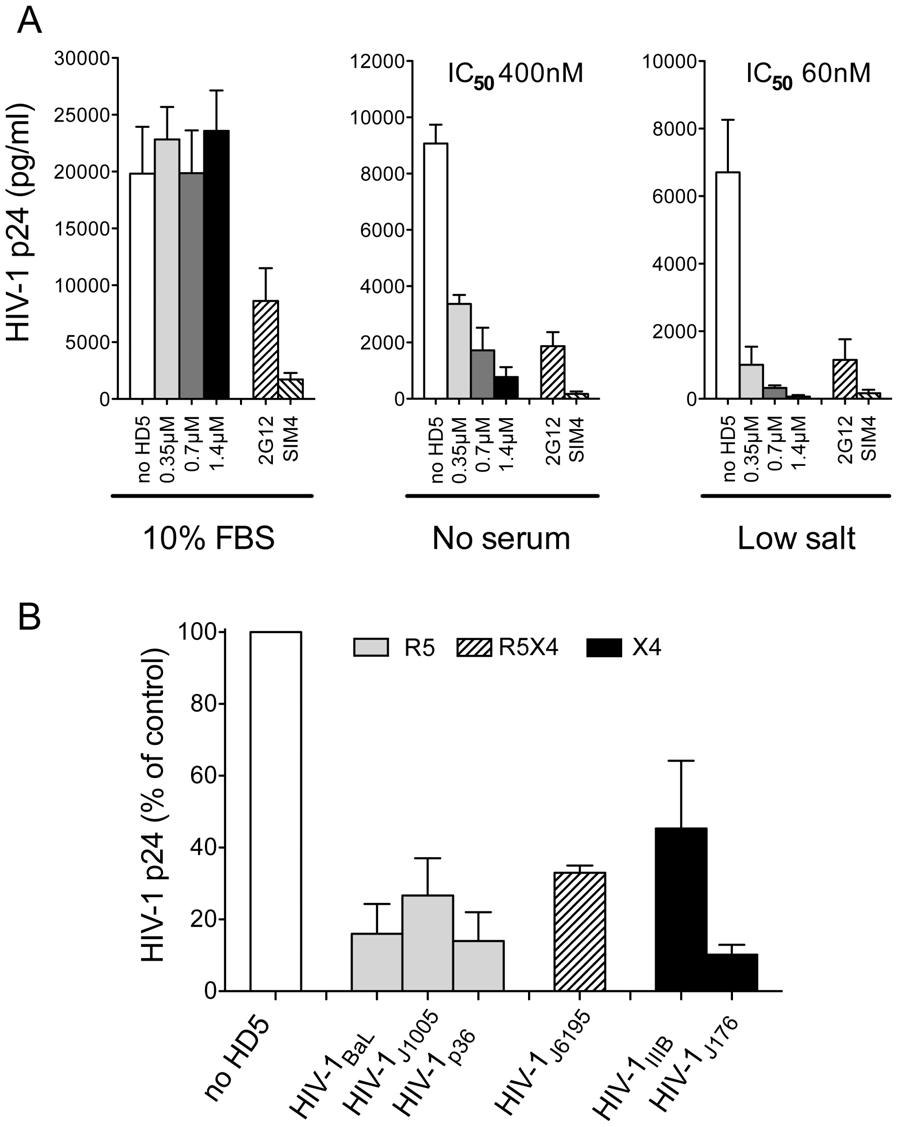
Effect of HD5 on HIV-1 replication in purified CD4^+^ T lymphocytes. (A) Effect of recombinant HD5 on infection by primary isolate HIV-1_J176_ in cell-free infection assays performed using the spinoculation method. HIV-1 virions were preincubated for 1 hour at 37°C with HD5 at the indicated concentrations in either RPMI-10 (10%FBS), RPMI-0.3-ITS (no serum) or 10 mM phosphate buffer 0.3-ITS (low salt). Gag p24 concentrations in the extracellular supernatant were measured on day 6 post-infection. Neutralizing mAbs directed to gp120 (2G12) or CD4 (Sim4) were used as positive controls to exclude non-specific infection. The data represent mean values (±SD) from 3 experiments each performed in triplicate. (B) Broad-spectrum inhibition of different HIV-1 strains by HD5. Purified CD4^+^ T lymphocytes were infected with two laboratory-adapted strains (HIV-1_IIIB_ and HIV-1_BaL_) or 4 primary clinical isolates (HIV-1_J1005_, HIV-1_p36_, HIV-1_J6195_ and HIV-1_J176_) in the presence of HD5 at 1.4 µM in RPMI-0.3-ITS. Gray bars indicate R5 HIV-1 isolates; solid bars X4 isolates; the striped bar a dualtropic isolate. The data represent mean values (±SD) of three separate experiments, each performed in triplicate.

Since the mucosal surfaces are a virtually serum-free environment, and several proteins present in bovine serum are known to inactivate α-defensins [19], [29], [30] we first focused on optimizing the culture conditions for infection of primary human CD4^+^ T cells in serum-free medium. In agreement with previous observations [31], the lack of serum proteins in the assay significantly decreased the infectivity of HIV-1 resulting in a reduction in virus entry from 30 to 70% depending on the HIV-1 strain used (data not shown). Therefore, to increase virus uptake by target cells we used the spinoculation method, which was reported to significantly improve the efficiency of infection [32]. Indeed, this method yielded a substantially higher level of infection compared to conventional static protocols (data not shown). Thus, we tested the ability of increasing concentrations of HD5 to inhibit infection by a primary HIV-1 isolate (HIV-1_J176_) in primary CD4^+^ T lymphocytes. As shown in Figure 1A, we found that HD5, in the absence of serum, exhibited a potent dose-dependent suppression of HIV-1 replication, with half-maximal inhibitory concentration (IC_50_) in the nanomolar range (400 nM). The broadly neutralizing mAbs 2G12 (gp120-specific) and Sim4 (CD4-specific) also inhibited infection, indicating that this infection protocol does not alter the physiological HIV-1 entry pathway mediated by envelope-receptor interaction by inducing non-specific membrane fusion events.

Next, we examined the effect of FBS on the antiviral activity of HD5. In agreement with previous observations [29], in the presence of serum HD5 failed to inhibit, and in some cases even slightly upregulated HIV-1 replication (Figure 1A). Since mucosal fluids typically have a low ionic strength [33] and the antimicrobial activities of HD5 are potentiated by low-salt media [34], we also explored the effect of ionic strength on HD5 activity. For this purpose, we infected CD4^+^ T lymphocytes with HIV-1_J176_ pre-incubated with HD5 in low-salt medium (10 mM phosphate buffer supplemented with 0.3% human AB serum, ITS supplement (Insulin, Transferrin, Sodium Selenite; Sigma) and 50 U/ml of rIL-2). Figure 1A shows that a low-salt environment decreased the amount of HD5 necessary to suppress viral replication (IC_50_, 60 nM). MAbs 2G12 and Sim4 also potently inhibited infection under low-salt conditions.

The breadth of anti-HIV activity of HD5 was investigated in activated CD4^+^ T lymphocytes using a panel of primary and laboratory-adapted HIV-1 isolates of different coreceptor-usage phenotype: three CCR5-specific (R5; HIV-1_BaL_, HIV-1_J1005_, HIV-1_p36_), two CXCR4-specific (X4; HIV-1_IIIB_, HIV-1_J176_) and one dual-tropic (R5X4; HIV-1_J6195_). HD5, used at 1.4 µM, inhibited the replication of all the HIV-1 isolates tested (Figure 1B). These data indicate that HD5 inhibits different HIV-1 isolates irrespective of their coreceptor-usage phenotype. Most importantly, HD5 is effective against primary HIV-1 isolates, which are physiologically relevant and generally more resistant to antibody-mediated neutralization.

### Human α-defensin-5 Inhibits HIV-1 Envelope-mediated Membrane Fusion

**Figure 2:**
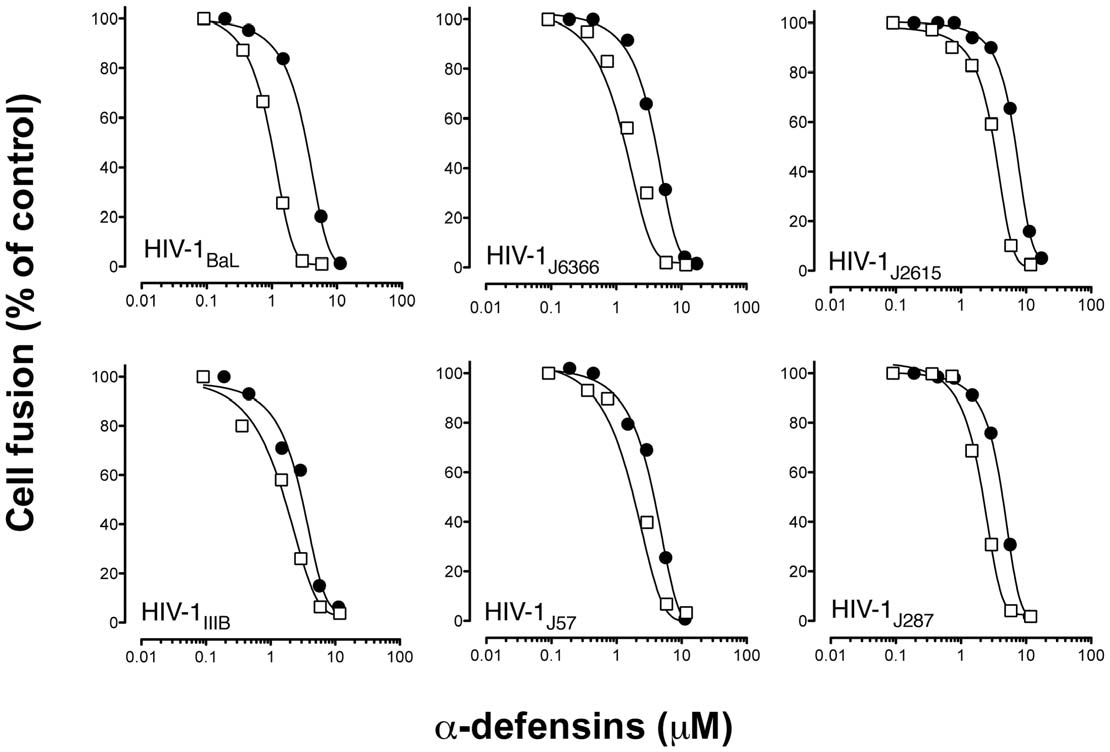
HD5 inhibits HIV-1 envelope-mediated fusion induced by clinical and laboratory HIV-1 isolates with different coreceptor usage. Fusion assays were performed using the human T-cell line (PM1) chronically infected with two prototypic laboratory-adapted strains (HIV-1_IIIB_ and HIV-1_BaL_) and four primary clinical isolates (R5: HIV-1_J6366_ and HIV-1_J2615_; X4: HIV-1_J57_ and HIV-1_J287_) cocultured with NIH-3T3 cells expressing membrane-bound CD4 and either CCR5 or CXCR4 in the presence or absence of increasing concentrations of HD5 (filled circles) for 2 hrs at 37°C in medium without FCS. For comparison, inhibition by HNP1 (open squares) used at the same concentrations is shown. The data are normalized with respect to fusion observed in the absence of inhibitors. The data represent mean values (±SD) from 3 experiments.

To understand if HD5, similarly to HNP1, blocks the early steps of the HIV-1 infectious cycle, we used a quantitative fusion assay based on vaccinia virus technology. PM1 cells expressing HIV-1 envelopes of two prototypic laboratory-adapted strains (IIIB, BaL) and four primary isolates (two R5, J6366 and J2615, and two X4, J57 and J287) were cocultured with NIH-3T3 cells expressing recombinant CD4 and either CCR5 or CXCR4 in the presence or absence of increasing concentrations of HD5. HNP-1, which was previously shown to inhibit HIV-1 envelope-mediated fusion [19], was used as a positive control. Figure 2 shows that HD5 inhibited fusion mediated by all the HIV-1 envelopes tested in a dose-dependent fashion. The inhibitory activity was independent of the envelope coreceptor specificity, since both R5 and X4 HIV-1 were inhibited alike even though the IC_50_ values for the R5 viruses used in these assays were slightly higher than those for the X4 isolates (mean IC_50_: 3.67±1,62 µM for R5 envelopes versus 2.77±0.38 µM for X4 envelopes). Consistent with the results obtained in cell-free HIV-1 infection assays, HD5 inhibited with comparable efficiency both laboratory-adapted and primary HIV-1 isolates and exhibited an inhibitory potency in the same range as that of HNP-1 (mean IC_50_ 2.01±0.69 µM). Because envelope-mediated cell fusion closely mimics the molecular events leading to viral entry, these results demonstrate that HD5 blocks HIV-1 infection at its earliest stages, before the viral entry step.

The effect of FBS on HD5 activity was also tested in the fusion assay. As seen in cell-free infection assays, concentrations of FBS as low as 2.5% vol/vol, reduced the inhibitory activity of HD5 by more than one log (IC_50_: 2.3 µM without FBS vs. 56 µM with FBS). An equivalent effect was observed in the presence of 2.5% normal human serum, whereas the levels of maximal fusion in absence of inhibitors were not influenced by the absence of serum in the assay medium (data not shown).

**Figure 3:**
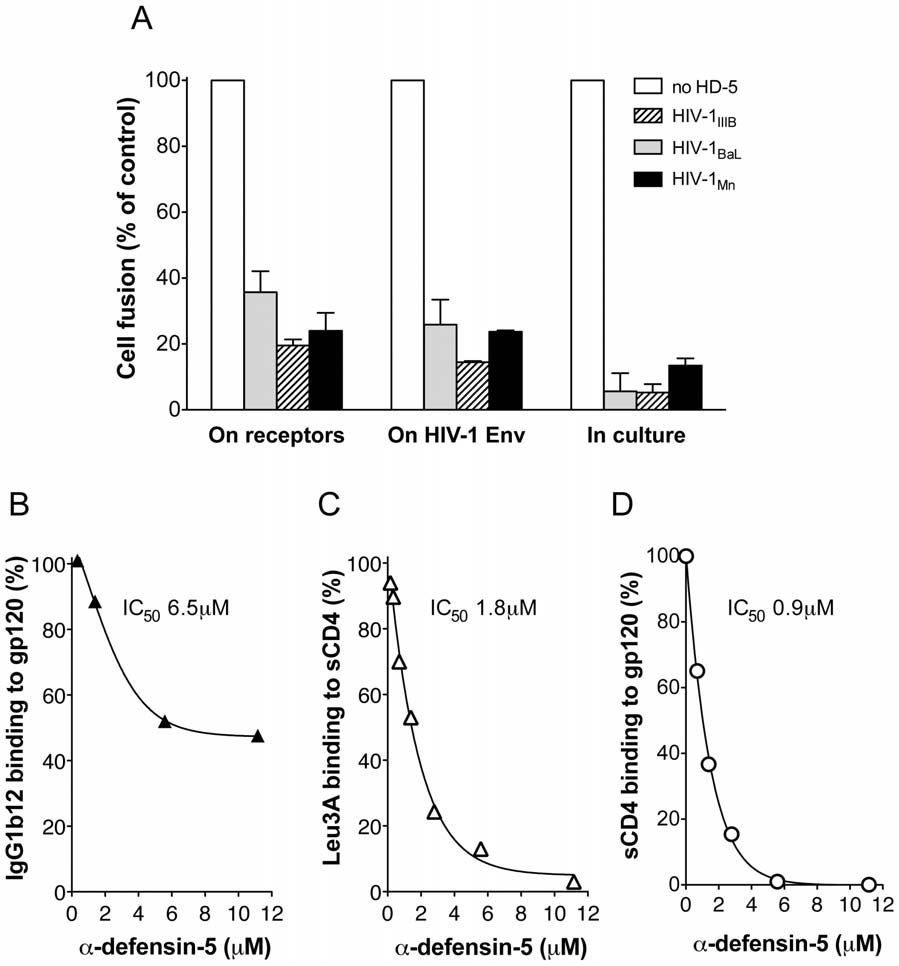
HD5 binds to both gp120 and CD4, and blocks the interaction of CD4 with gp120. (A) Effect of HD5 on HIV-1 envelope-mediated fusion after pre-incubation with effector cells (expressing the viral envelope) or target cells (expressing CD4 and the coreceptor). Prior to the fusion reaction, HD5 was pre-incubated for 20 minutes with effector cells or target cells followed by washing to remove the unbound defensin. Control fusion was performed as in Figure 2 with addition of HD5 at the time of culture between effectors and targets. (B) Binding of HD5 to HIV-1 gp120. Competition of synthetic HD5 with the anti-gp120 mAb IgG1b12 (used at 5 µg/mL) for binding to plastic-immobilized recombinant gp120_BaL_ in ELISA. (C) Binding of HD5 to human CD4. Competition of synthetic HD5 with the anti-CD4 mAb Leu3a for binding to plastic-immobilized recombinant sCD4 in ELISA. The plates were coated with sCD4 at 5 µg/mL; HD5 was added prior to Leu3a and kept in the wells throughout the reaction period. (D) Inhibition of gp120/CD4 binding by HD5. Competition of HD5 with recombinant HIV-1 gp120_BaL_ for binding to plastic-immobilized sCD4 in ELISA. The plates were coated with sCD4 at 5 µg/mL; HD5 was added prior to Leu3a or gp120_BaL_ and kept in the wells throughout the reaction period. Error bars indicate SD of mean values obtained from 3 repeated assays.

To gain insight into the mechanism(s) of inhibition of HIV-1 envelope-mediated fusion by HD5, we tested the effect of HD5 pre-treatment of either effector cells (expressing the HIV-1 envelope) or target cells (expressing CD4 and the coreceptor) followed by removal of the inhibitor during the fusion assay. Three different viral strains were used for this purpose: two X4 (HIV-1_MN_ and HIV-1_IIIB_) and one R5 (HIV-1_BaL_). HD5 was pre-incubated with either the effector or the target cells for 20 minutes at 37°C and then removed before initiation of the fusion reaction. As a control, HD5 was regularly added at the time of effector-target cell mixing and maintained in the culture medium throughout the fusion reaction period. As illustrated in Figure 3A, HD5 was able to inhibit fusion whether pre-incubated with the effector cells or with the target cells. For two of the HIV-1 envelopes tested, the inhibition was slightly more pronounced when HD5 was incubated with envelope-expressing cells. These data suggest that HD5 directly interacts with both the viral envelope and the cellular receptor molecules, in line with our previous observations with α-defensins-1 and -2 [19].

### Specific Binding of α-defensin-5 to HIV-1 gp120 and to Human CD4

Next, we evaluated whether HD5 was able to bind directly to the molecules involved in the first HIV-1 docking event on the target cell surface: gp120 and CD4. We previously demonstrated that α-defensins-1 and -2 compete with the binding of a gp120-specific monoclonal antibody, IgG1b12, to recombinant HIV-1 gp120 [19]. Thus, we tested the ability of increasing concentration of HD5 to compete with IgG1b12 in ELISA. As shown in Figure 3B, HD5 reduced in a dose-dependent fashion binding of IgG1b12 to gp120_SF162_, with an IC_50_ of 6.5 µM, demonstrating a direct interaction of HD5 with gp120. However, the competition was not complete even at the highest doses of HD5, suggesting an incomplete epitope overlap between the antibody- and the HD5-binding regions of gp120.

To investigate whether HD5 binds to CD4, the primary cellular receptor for HIV-1, increasing amounts of HD5 were used in ELISA to compete with the binding of a CD4-specific mAb, Leu3a, to a recombinant, truncated form of soluble CD4 (sCD4) immobilized on plastic. As shown in Figure 3C a dose-dependent inhibition of Leu3a binding was observed, with an IC_50_ of 1.8 µM, demonstrating that HD5 directly interacts with the CD4 molecule. Interestingly, the Leu3a-binding site maps to a region within the D1–D2 domain of CD4 that is directly involved in gp120 binding and inhibits gp120 binding and HIV-1 infection *in vitro*. In contrast, no inhibitory effect was seen when the same competition assays was repeated using mAb OKT4 (not shown), which recognizes an epitope within the D3 and D4 domains, both dispensable for gp120 binding. These results demonstrated that HD5 interacts with the D1–D2 domains of CD4, thus potentially hindering gp120 access to its binding site.

### α-defensin-5 Inhibits Binding of gp120 to CD4

The demonstration that HD5 blocks HIV-1 envelope-mediated fusion and directly interacts with both HIV-1 gp120 and CD4 did not provide formal proof that HD5 effectively interferes with gp120/CD4 binding. Therefore, we directly evaluated the ability of HD5 to compete with binding of recombinant soluble CD4 to plastic-immobilized recombinant gp120. Figure 3D shows that HD5 efficiently competes with the binding of sCD4 to gp120. Of note, the IC_50_ value for this competition (0.9 µM) was lower than that obtained for HD5 competition with anti-gp120 and anti-CD4 antibodies on the corresponding molecules (6.5 and 1.8 µM, respectively), indicating an additive inhibitory effect that is consistent with HD5 binding to both sides of the gp120-CD4 interface.

### Mapping of the HD5-binding Region of gp120

**Figure 4:**
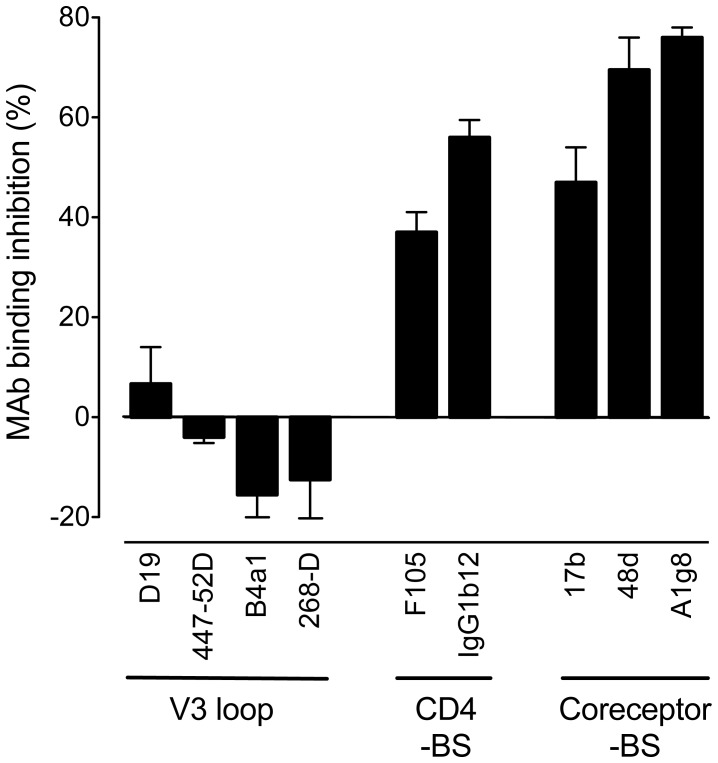
Mapping of the HD5-binding site in HIV-1 gp120. Competition of HD5 with a panel of anti-gp120 mAbs with various epitope specificity for binding to plastic-immobilized recombinant gp120 in ELISA. The plates were coated with gp120_BaL_ at 2 µg/mL; HD5 at 5.6 µM was added prior to the anti-gp120 mAbs, all used at 5 µg/ml. CD4 binding site (CD4-BS), CD4-induced coreceptor binding site (Coreceptor-BS). The data represent mean values (±SD) from three experiments.

To define the putative binding region of HD5 within the gp120 glycoprotein, we used a panel of gp120-specific mAbs of human or murine origin that recognize three major functional domains of gp120: the CD4-binding site, the coreceptor-binding site and the V3 loop. ELISA assays were performed using recombinant monomeric gp120 immobilized on plastic. A decreased binding activity was seen with mAbs directed against the CD4-binding region (F105, IgG1b12), or against CD4-induced epitopes that overlap with the coreceptor-binding site (17b, 48d and A1g8); by contrast, no significant interference was seen with the binding of mAbs 447-52D, B4a1 and 268-D, all directed against relatively conserved epitopes within the V3 domain (Figure 4). These data confirmed that HD5 directly interacts with HIV-1 gp120, binding to a region that overlaps, is contiguous to, or influences the conformation of the CD4- and coreceptor-binding domains. Similar results were obtained using a second recombinant gp120, derived from another R5 isolate, HIV-1_SF162_ (data not shown).

### Differential Effect of HD5 on HIV-1 Coreceptor Expression

**Figure 5:**
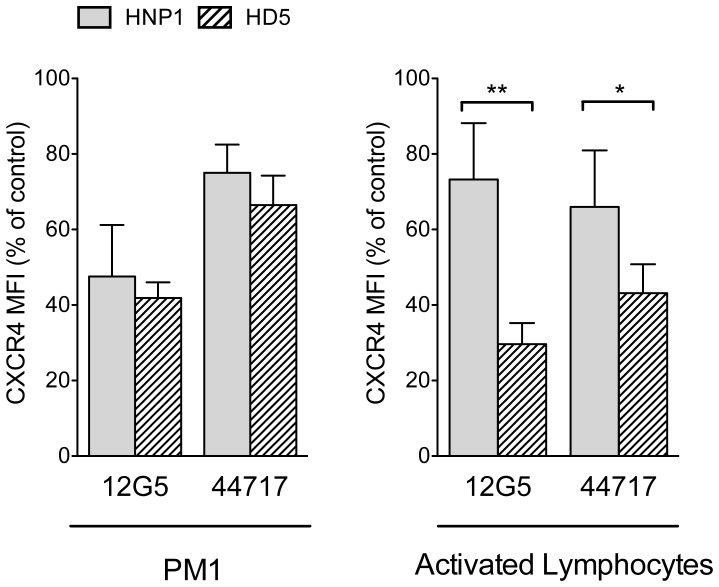
Effect of HD5 on cell-surface HIV-1 coreceptor expression: induction of CXCR4 downmodulation. PM1 cells and primary peripheral blood lymphocytes activated with PHA and IL-2 were preincubated for 2 hours with or without HNP1 or HD5 (each at 14.7 µM) in RPMI-ITS without serum; the cells were then stained with 12G5 or 44717.11, two_conformation-dependent mAbs that recognize antigenically distinct populations of CXCR4 [39] and analyzed by flow cytometry. The level of expression was determined by differences between the level of staining with specific mAbs and with the isotype control. The results are expressed as percent reduction of mean fluorescent intensity (MFI) compared to untreated controls. Data are representative of 4 to 6 independent experiments performed with similar results. Error bars show standard deviations from the mean. *, p = 0.032 and **, p = 0.0084. Wilcoxon Mann-Whitney rank-sum test, 2-tailed.

After binding to CD4, gp120 engages a coreceptor, such as CXCR4 or CCR5, in order to promote viral entry into susceptible cells [35]. We questioned if the antiviral activity of HD5 could be in part mediated by interaction with the coreceptors. The effect of HD5 on coreceptor expression was studied by flow cytometry both on PM1 cells, which naturally express both CCR5 and CXCR4 [36], and on activated peripheral blood lymphocytes, which represent the most physiological *ex vivo* model. The activity of HD5 was compared with that of HNP1. Target cells were incubated with or without HD5 or HNP1 at 14.7 µM in RPMI-0 for 2 hours at 37C° and then stained with two anti-CXCR4 mAbs that inhibit infection by X4 HIV-1 strains [37]: 12G5, a conformation dependent mAb directed against a bridging epitope spanning the first and second extracellular loops of CXCR4 [38], and 44717.111, a conformation-dependent mAb that recognizes CXCR4 with higher efficiency that 12G5 on both PM1 and primary T lymphocytes [39]. Figure 5 shows that HD5 inhibited the binding of both 12G5 (mean fluorescence intensity [MFI] reduction 58.5±2.9% on PM1; 70.04% ±2.8% on primary T cells) and 44717.111 (MFI reduction 33.5±5.5% on PM1; 56.9% ±3.1% on primary T cells). Interestingly, in the PM1 cells model HD5 was slightly more effective than HNP1 in reducing CXCR4 expression, while in primary T cells HD5-induced downmodulation of CXCR4 expression was significantly more pronounced. However, neither protein had any effect on CXCR4 expression when used at concentrations below 3.5 µM (data not shown).

In contrast to CXCR4, the surface expression of CCR5, measured with a panel of different mAbs directed against different epitopes of CCR5, was not reduced upon treatment with HD5 or HNP1 (data not shown). Incubation of paraformaldehyde-fixed cells before staining with mAbs did not alter the detection of CCR5 or CXCR4 (data not shown), indicating that antibody binding was not directly blocked by defensins and confirming that the observed effect on CXCR4 expression was due to receptor modulation.

## Discussion

Human α-defensins are effectors of innate immunity produced by leukocytes (α-defensins 1–4) or by specialized epithelial cells (α-defensin 5–6), which exert a broad-spectrum antimicrobial activity [40]. In particular, HD5 was shown to inhibit two viruses with genital tropism, herpes simplex virus and papillomavirus [24], [41], but its effect on HIV-1 was not clearly established. Using different assays, previous studies observed either a lack of effect or even an enhancing effect of HD5 on HIV-1 replication [27], [28], [42]. However, these assays were performed in the presence of bovine serum, which is a non-physiological substance that is known to alter the immunological [43], [44], antibacterial [34], and antiviral [24], [29] functions of defensins. Indeed, we confirmed that in presence of bovine serum, HD5 had no effects or even slightly upregulated HIV-1 replication in primary CD4^+^ T lymphocytes, but the physiological significance of the latter finding is questionable. HD5 is naturally secreted at mucosal surfaces [23], which constitute a virtually serum-free environment. We decided to address this issue by optimizing the culture conditions for testing HIV-1 infection and fusion in the presence of only trace amounts or in complete absence of serum-derived proteins. Using these assays, we were able to demonstrate that HD5 is a potent and broad-spectrum inhibitor of biologically diverse HIV-1 strains. Indeed, the inhibitory activity of HD5 was independent of the HIV-1 coreceptor-usage phenotype and was seen not only on laboratory-adapted strains but also on primary clinical isolates minimally passaged *ex vivo*. Moreover, the inhibition occurred at concentrations in the low micromolar range similar to those effective against papillomavirus and adenovirus [24], [26], [29]. Another peculiar feature of the genital mucosae is that they are characterized by low-salt concentrations [33]. To mimic these conditions, we tested the effect of HD5 in low-ionic strength buffer and documented an even stronger antiviral activity at nanomolar levels that are those commonly found in the male and female lower genital tracts [23], [45]. These results suggest that the antiviral potency and mechanism of action of defensins at mucosal surfaces may be different than in other anatomical sites in the body.

Another important variable in the study of defensins is represented by the concentrations used in *in vitro* models versus those reached in specific organs *in vivo*. It has been reported that high levels of HD5 (20 to 50 µg/ml) moderately enhance the replication of primary HIV-1 strains and strongly enhance the replication of pseudotyped HIV-1 luciferase reporter viruses [27], [28]. In fact, it is conceivable that at such high concentrations HD5 forms polycationic aggregates that enhance HIV-1 infectivity by neutralizing membrane charges and/or by promoting virus aggregation [46], [47]. A similar phenomenon has been described for RANTES, a CCR5-binding chemokine that otherwise, at physiological concentrations, is a potent and specific inhibitor of HIV-1 [48]. Furthermore, firefly luciferase, used in most of these studies [27], [28], has been recently shown to be inhibited by defensins, hampering the interpretation of these data [20].

In this report, we identified one mechanism of HD5-mediated HIV-1 inhibition, demonstrating that HD5 directly interacts both with the major HIV-1 envelope glycoprotein, gp120, and with its primary cellular receptor, CD4, interfering with their reciprocal binding. Thus, HD5 inhibits HIV-1 infection at a very early stage, before viral entry. These observations are in line with the mechanisms of action that we previously demonstrated for α-defensins-1 and -2 [19], which share both sequence and structural homology with HD5. In spite of these similarities, the anti-HIV activity of HD5 was not an obvious finding. HD5 shares with α-defensin 1–3 only 37% amino acid identity and has divergent antimicrobial properties [14], [16]. The ability of α-defensins 1, 2 and 5 to bind specifically to both gp120 and CD4 is also consistent with the mechanism of action of θ-defensins [30], which also function as HIV-inhibitors. The capacity of most defensins to bind different proteins may depend on electrostatic interactions mediated by the high concentration of surface positive charges that characterizes this family of peptides [14] and underlies their lectin-like activity [30]. Both CD4 and gp120 are heavily glycosylated proteins. Nevertheless, we found that HD5 and α-defensins-1 and -2 possess distinct binding specificities. We demonstrated that HD5 effectively competes with mAb Leu3a that recognizes the D1 region of CD4, which includes the gp120-binding site [49] but not with OKT4 that is directed to the D3 and D4 domains. On the gp120 side, we found competition with mAbs mapping to the CD4- and coreceptor-binding sites, but not with mAbs to the V3 loop. The lack of interaction with the V3 loop is consistent with the positively-charged nature of this region of gp120 [50]. Moreover, the V3 loop sequence and charge determine the coreceptor usage [51] and this is consistent with the broad anti-HIV-1 activity and the lack of preferential interaction of HD5 and HNP-1 [19], [20] with R5 vs. X4 HIV-1 variants. Both CCR5 and CXCR4 coreceptors are glycosylated molecules that play a crucial role in the process of viral entry. Thus, we postulated they could bind HD5 as previously shown for other α- and β-defensins [19], [20], [21], [52], [53]. We found that HD5 downmodulated the expression of CXCR4 but not of CCR5. Interestingly, HD5 was significantly more efficient than HNP1 in downmodulating CXCR4 in activated primary T cells, which are the principal target of HIV-1 infection. Decreased cell surface CXCR4 has been reported to result in marked inhibition of X4 HIV-1-mediated fusion efficiency [54] and viral replication [55]; thus, CXCR4 modulation represents another possible mechanism of HD5-mediated HIV-1 inhibition at the entry level. However, consistently with previous data on HNP1 [19], [20] inhibition of cell-free infection by HD5 required much lower concentrations of the defensin and did not discriminate between X4 and R5 viral strains. Thus, we can speculate that coreceptor modulation is not the primary mechanism of HIV-1 inhibition by HD5 at the level of genital mucosae although it could play a more important role within the intestinal mucosa where HD5 can reach concentrations in the order of 90–450 µg/cm^2^ [56].

The HIV-1 gp120 envelope glycoprotein contains a number of features that help it evade humoral immunity, including the positioning of the highly conserved CD4-binding site in regions poorly accessible to neutralizing antibodies and the presence of multiple N-linked glycosylation sites accounting for over one-half of the molecular mass [57]. HD5 posses both a strong capacity to bind carbohydrates and glycoproteins including gp120_IIIB_ (K_d_ 24.5 nM) and gp120_BaL_ (K_d_108 nM) [47], and being a very small and tightly folded molecule it is likely to have access to hidden neutralization sites where full-length antibodies may not be able to reach.

Although our results indicate that HD5 inhibits the earliest stages of HIV-1 infection, preventing viral entry into the target cell, we cannot exclude an effect on downstream steps in the virus life cycle, as previously shown for HNP-1 [20], [58]. Interestingly, it was demonstrated that HD5 and HNPs inhibit herpes simplex virus infection by acting both at the entry and post-entry levels [41]. A growing body of evidence [17], [20], [21] is compatible with the notion that multiple mechanisms cooperate toward inhibition of HIV-1 infection by human defensins, and further studies will be required to define these complex mechanisms of viral inhibition.

HD5 is expressed and stored in secretory granules of the Paneth cells located at the base of the crypts in the small intestine. The massive HIV-1 replication that takes place in the gastrointestinal tract early in the course of the infection not only plays a critical role in the massive depletion of CD4^+^ T cells within the lamina propria, but also results in a seemingly irreversible damage to the architecture of the intestinal mucosa leading to crypt hyperplasia, decreased number of Paneth cells and decreased levels of luminal defensins [59], [60]. These observations, together with our data on the antiviral effect of HD5 suggest a possible role of HD5 in the pathogenesis of HIV-1 infection, which needs to be addressed in further studies.

Our data also show that HD5 inhibits HIV-1 infection in primary CD4^+^ T lymphocytes at concentrations similar to those measured in the female and male lower genital tracts [23], [45], suggesting that HD5 can function as a natural immune barrier against HIV-1 sexual transmission. Moreover, HD5 may share the mechanism of action of cyanovirin-N, a lectin-like protein that binds to mannose moieties in gp120 and of mAb IgG1b12, with which it competes for the same binding site on CD4 receptor. Both of these virus-targeting entry-inhibitors have been reported to protect macaques from vaginal SHIV-challenge [61].

A potential exploitation of the antiviral properties of HD5 could be the exogenous application of synthetic defensin peptides as part of a preventive microbicide strategy. So far, it has been difficult to establish an association *in vivo* between levels of mucosal antimicrobial peptides and decreased HIV-transmission [62] since higher genital levels of anti-HIV immune factors are invariably associated with bacterial vaginosis and sexually transmitted infections, which are known to increase HIV susceptibility [63]. However, the prospect that HD5 might function as a safe, nonimmunogenic and noninflammatory topical microbicide is supported by the fact that this defensin is normally found at antiviral concentrations in the female genital tract [23], [45]. Moreover, the documented antimicrobial effect of HD5 against herpes simplex viruses, papillomavirus and pathogenic bacteria like *Neisseria gonorrhoeae* and *Chlamydia trachomatis*, all of which cause genital infections that act as cofactors in HIV-1 transmission, suggests that this defensin has the potential to function as a broad-spectrum topical microbicide [64]. Pre-clinical studies in suitable animal models will be important to assess the potential efficacy of HD5 in the prevention and control of HIV-1 infection.

## Methods

### Proteins and Antibodies

Synthetic human HD5 was purchased from Peptides International (Louisville, KY). Recombinant HIV-1 gp120 proteins from HIV-1 isolates SF162 and BaL, recombinant human soluble CD4 (sCD4), human anti-HIV-1 gp120 mAb directed to the V3 loop (447-52D, B4a1 and 268-D), to the CD4-binding site (F105 and IgG1b12) and to CD4-induced epitopes adjacent to the coreceptor-binding site (17b, 48d and A1g8) and anti-CXCR4 mAb 12G5 were obtained from the NIH AIDS Research and Reference Reagent Program (ARRRP, Rockville, MD). Murine anti-V3 loop mAb D19 was obtained by standard hybridoma technology as previously described [65]. Additional mAbs used were anti-CD4, Leu3a (Becton Dickinson, San Jose, CA) and OKT4 (Ortho Diagnostics, Raritan, NJ), anti-CCR5 clone 2D7 (BD PharMingen, La Jolla, CA), anti-CXCR4 mAb clone 44717.111 (R&D Systems, Minneapolis, MN), anti-CD8, -CD14, -CD19 and -CD56 (ImmunoTools, Friesoythe, Germany).

### Viruses and Persistently Infected Cell Lines

Laboratory-passaged HIV-1 isolates IIIB, MN and BaL were obtained through the NIH ARRRP. All the primary HIV-1 isolates, kindly provided by Dr. Gabriella Scarlatti (DIBIT-HSR, Milan), were obtained by cocultivation of patient peripheral blood mononuclear cells (PBMC) with activated PBMC from healthy blood donors and minimally passaged *in vitro* exclusively in primary cells, as previously reported [66]. Infectious viral supernatants were used to prepare chronically infected PM1 and SupT1 as previously described [65]. Serum-free virus stocks were prepared by ultracentrifugation of the viral stocks, originally produced in the presence of 10% FBS, on a 20% sucrose cushion as described [67], with slight modifications. Briefly, cell debris was initially removed by centrifugation at 470×g for 5 min and subsequent filtration through a 0.22-µm pore-size filter. The virus stocks were layered on top of a 20% (wt/vol) sucrose solution in TNE buffer (20 mM Tris [pH 8.0], 150 mM NaCl, and 2 mM EDTA). The virus was pelleted at 19,000×g for 2 h at 4°C and resuspended in serum-free RPMI supplemented with glutamine and antibiotics (RPMI-0). Viral stocks were titrated on activated human CD4^+^ T cells. Aliquots were frozen at −80°C. HeLa and SupT1 cell lines were obtained from the American Type Culture Collection. The PM1 cell clone and NIH3T3 mouse fibroblasts stably coexpressing human CD4 and either CCR5 or CXCR4 were provided by the NIH ARRRP. The study protocol was approved by the institutional review board at the San Raffaele Research Institute (Milan, Italy). Written consent was obtained from all study participants.

### Acute HIV-1 Infection Assay

PBMCs were isolated from healthy blood donors by standard Ficoll-HyPaque (Pharmacia, Uppsala, Sweden) density gradient centrifugation from concentrated leukocytes of healthy blood donors and stimulated with 5 µg/ml PHA (Sigma, St. Louis, MO) in complete RPMI medium supplemented with 10% FBS and 50 U/ml recombinant IL-2 (Proleukin; Chiron Corporation, Emeryville, CA) (RPMI-10). After 3 days in culture, CD4^+^ T cell population was enriched by negative immunomagnetic selection using Dynabeads M-450 (Invitrogen Carlsbad, CA) coated with monoclonal antibodies to CD8, CD14, CD19 and CD56 (Sigma, St. Louis, MO) according to the manufacturer’s protocol. Infection was carried out by spinoculation for 1.5 hours as previously described [32] with minor modifications. Briefly, CD4^+^ T cells were washed twice in RPMI-0 and plated (10^5^/well) in 96-wells U-bottom microtiter plates with 50 50% tissue culture infective dose (TCID_50_) of serum-free HIV-1 virions, preincubated for 1 hour at 37°C with HD5 at the indicated concentrations. The plates were centrifuged at 1,000×g for 1.5 hrs and washed twice with RPMI-0 to remove unbound virus, after which HD5 was added back to the cells. Residual virus associated to cells after washes was below 100pg of p24/ml (day 0 time point). Infected cells were cultured in 200 µl of RPMI-1640 supplemented with 0.3% human AB serum, ITS supplement (Insulin, Transferrin, Sodium Selenite; Sigma) and 50 U/ml of rIL-2 (RPMI-0.3-ITS) in the presence or absence of inhibitors. Culture in ITS serum-free media supplement and 0.3% human AB serum to RPMI-0 culture medium did not significantly alter the viability of purified CD4^+^ lymphocytes or PM1 and SupT1 cell lines, as analyzed using the CellTiter 96 Aqueous One Solution Cell Proliferation Assay kit (Promega Madison, WI), nor the expression of CD4 and the CCR5 and CXCR4 coreceptors, as analyzed by flow cytometry as previously described [19] (data not shown).

The HIV-1 p24 Ag concentrations in the culture supernatants were determined by enzyme-linked immunosorbent assay (ELISA) at days 4 and 6 post-infection as previously described [68]. Briefly, p24 antigen from a detergent lysate of virions was captured by an immobilized anti-p24 polyclonal antibody (D7320; Aalto Bio Reagents, Dublin, Ireland). Bound p24 Ag was then detected using an alkaline phosphatase–conjugated anti-p24 monoclonal antibody (BC 1071-AP; Aalto Bio Reagents) and the AMPAK ELISA amplification system (DAKO A/S, Glostrup, Denmark).

### HIV-1 Envelope-mediated Cell Fusion Assay

The HIV-1 Env-mediated fusion assay was performed as described [65] using a modification of the test originally developed by Berger and coworkers [69].

In the modified assay, HIV-1 Env effector cells were T cell lines (PM1 or SupT1) chronically infected with laboratory strains or primary HIV-1 isolates, (coinfected with recombinant vaccinia virus vCB21R encoding the *E Coli lacZ* reporter gene linked to the T7 promoter). Target cells were NIH 3T3 mouse fibroblast cells stably expressing human CCR5 or CXCR4 and human CD4 (infected with vaccinia recombinant vT7 encoding bacteriophage T7 RNA polymerase, vP11T7). Effector and target cells were mixed in 96-well plates (10^5^ each cell type per well) and incubated at 37°C in RPMI-0 with or without inhibitors; after 2 h cells were lysed with a nonionic detergent, and β-galactosidase activity was quantitated by spectrophotometry.

### ELISA Assays

Flat-bottom, 96-well MaxiSorp plates (NUNC, Roskilde, Denmark) were coated with recombinant sCD4 (5 µg/well) or HIV-1_BaL_ gp120 (2 µg/well) in phosphate-buffered saline (PBS) for 18 hrs at 4°C. Bovine serum albumin (BSA) 3% was used for blocking the plates. Antibodies were added in 100 µl PBS+0.05%BSA and incubated for 1 hr at 37°C, after which the plate was washed and incubated for an additional hour at room temperature with the secondary antibody. Peroxidase-conjugated, affinity-purified goat-anti-mouse IgG or goat-anti-human IgG (Dako, Glostrup, Denmark) were used as secondary antibodies. The reaction was revealed by using an appropriate substrate. The specific signal was calculated by subtracting background values obtained from replicate wells containing all the reagents except the specific ligand for each assay. Binding of mAbs 17b and 48d, directed to CD4-induced epitopes, was performed after preincubation of immobilized gp120 with sCD4 (2 µg/well) in PBS for 20 min at 37°C [65].

### Flow Cytometry

PM1 or PHA-activated peripheral blood lymphocytes were incubated with or without α-defensins in RPMI without FBS and then stained with the mAbs for 20 minutes at room temperature. Cells were then washed with PBS supplemented with 1% FBS and labeled with PE-conjugated polyclonal goat antimouse antibody (Sigma, St. Louis, MO). Cells treated with an irrelevant, isotype-matched mAb were used as negative controls. Samples were analyzed on a FACScan cytometer (Becton Dickinson, San Jose, CA). In some experiments, the cells were fixed with 2% paraformaldehyde before treatment with α-defensins and mAb staining.

## References

[1] WHO: AIDS Epidemic update 2010. UNAIDS website. Available: http://www.unaids.org/globalreport/. Accessed 2012 August 31.

[2] Fauci AS, Johnston MI, Dieffenbach CW, Burton DR, Hammer SM, et al (2008) HIV vaccine research: the way forward. Science 321: 530–532. 18653883 10.1126/science.1161000

[3] Shattock RJ, Haynes BF, Pulendran B, Flores J, Esparza J (2008) Improving defences at the portal of HIV entry: mucosal and innate immunity. PLoS Med 5: e81. 18384232 10.1371/journal.pmed.0050081 PMC2276525

[4] Sodora DL, Gettie A, Miller CJ, Marx PA (1998) Vaginal transmission of SIV: assessing infectivity and hormonal influences in macaques inoculated with cell-free and cell-associated viral stocks. AIDS Res Human Retrov 14 Suppl 1S119–123. 9581895

[5] Wawer MJ, Gray RH, Sewankambo NK, Serwadda D, Li X, et al (2005) Rates of HIV-1 transmission per coital act, by stage of HIV-1 infection, in Rakai, Uganda. J Infect Dis 191: 1403–1409. 15809897 10.1086/429411

[6] Hickey DK, Patel MV, Fahey JV, Wira CR (2011) Innate and adaptive immunity at mucosal surfaces of the female reproductive tract: stratification and integration of immune protection against the transmission of sexually transmitted infections. J Reprod Immunol 88: 185–194. 21353708 10.1016/j.jri.2011.01.005 PMC3094911

[7] Venkataraman N, Cole AL, Svoboda P, Pohl J, Cole AM (2005) Cationic polypeptides are required for anti-HIV-1 activity of human vaginal fluid. J Immunol 175: 7560–7567. 16301665 10.4049/jimmunol.175.11.7560

[8] Cole AM, Cole AL (2008) Antimicrobial Polypeptides are Key Anti-HIV-1 Effector Molecules of Cervicovaginal Host Defense. Am J Reprod Immunol 59: 27–34. 18154593 10.1111/j.1600-0897.2007.00561.x

[9] Wira CR, Patel MV, Ghosh M, Mukura L, Fahey JV (2011) Innate immunity in the human female reproductive tract: endocrine regulation of endogenous antimicrobial protection against HIV and other sexually transmitted infections. Am J Reprod Immunol 65: 196–211. 21294805 10.1111/j.1600-0897.2011.00970.x PMC3837338

[10] Valore EV, Park CH, Igreti SL, Ganz T (2002) Antimicrobial components of vaginal fluid. Am J Obstet Gynecol 187: 561–568. 12237628 10.1067/mob.2002.125280

[11] Levinson P, Choi RY, Cole AL, Hirbod T, Rhedin S, et al (2012) HIV-Neutralizing Activity of Cationic Polypeptides in Cervicovaginal Secretions of Women in HIV-Serodiscordant Relationships. PLoS One 7: e31996. 22389677 10.1371/journal.pone.0031996 PMC3289637

[12] Levinson P, Kaul R, Kimani J, Ngugi E, Moses S, et al (2009) Levels of innate immune factors in genital fluids: association of α-defensins and LL-37 with genital infections and increased HIV acquisition. AIDS 23: 309–317. 19114868 10.1097/QAD.0b013e328321809c

[13] Hill CP, Yee J, Selsted ME, Eisenberg D (1991) Crystal structure of defensin HNP-3, an amphiphilic dimer: mechanisms of membrane permeabilization. Science 251: 1481–1485. 2006422 10.1126/science.2006422

[14] Szyk A, Wu Z, Tucker K, Yang D, Lu W, et al (2006) Crystal structures of human α-defensins HNP4, HD5, and HD6. Protein Sci 15: 2749–2760. 17088326 10.1110/ps.062336606 PMC2242434

[15] Lehrer RI, Lu W (2012) α-Defensins in human innate immunity. Immunol Rev 245: 84–112. 22168415 10.1111/j.1600-065X.2011.01082.x

[16] Yang D, Biragyn A, Hoover DM, Lubkowski J, Oppenheim JJ (2004) Multiple roles of antimicrobial defensins, cathelicidins, and eosinophil-derived neurotoxin in host defense. Annu Rev Immunol 22: 181–215. 15032578 10.1146/annurev.immunol.22.012703.104603

[17] Klotman ME, Chang TL (2006) Defensins in innate antiviral immunity. Nat Rev Immunol 6: 447–456. 16724099 10.1038/nri1860

[18] Buck CB (2008) Defensins’ offensive play: exploiting a viral achilles’ heel. Cell Host Microbe 3: 3–4. 18191786 10.1016/j.chom.2007.12.006

[19] Furci L, Sironi F, Tolazzi M, Vassena L, Lusso P (2007) α-defensins block the early steps of HIV-1 infection: interference with the binding of gp120 to CD4. Blood 109: 2928–2935. 17132727 10.1182/blood-2006-05-024489

[20] Seidel A, Ye Y, de Armas LR, Soto M, Yarosh W, et al (2010) Cyclic and acyclic defensins inhibit human immunodeficiency virus type-1 replication by different mechanisms. PLoS One 5: e9737. 20305815 10.1371/journal.pone.0009737 PMC2840026

[21] Demirkhanyan LH, Marin M, Padilla-Parra S, Zhan C, Miyauchi K, et al (2012) Multifaceted mechanisms of HIV-1 entry inhibition by human α-defensin. J Biol Chem 287: 28821–28831. 22733823 10.1074/jbc.M112.375949 PMC3436536

[22] Svinarich DM, Wolf NA, Gomez R, Gonik B, Romero R (1997) Detection of human defensin 5 in reproductive tissues. Am J Obstet Gynecol 176: 470–475. 9065200 10.1016/s0002-9378(97)70517-9

[23] Quayle AJ, Porter EM, Nussbaum AA, Wang YM, Brabec C, et al (1998) Gene expression, immunolocalization, and secretion of human defensin-5 in human female reproductive tract. Am J Pathol 152: 1247–1258. 9588893 PMC1858596

[24] Buck CB, Day PM, Thompson CD, Lubkowski J, Lu W, et al (2006) Human α-defensins block papillomavirus infection. Proc Natl Acad Sci U S A 103: 1516–1521. 16432216 10.1073/pnas.0508033103 PMC1360544

[25] Dugan AS, Maginnis MS, Jordan JA, Gasparovic ML, Manley K, et al (2008) Human α-Defensins Inhibit BK Virus Infection by Aggregating Virions and Blocking Binding to Host Cells. J Biol Chem 283: 31125–31132. 18782756 10.1074/jbc.M805902200 PMC2576552

[26] Smith JG, Silvestry M, Lindert S, Lu W, Nemerow GR, et al (2010) Insight into the mechanisms of adenovirus capsid disassembly from studies of defensin neutralization. PLoS Pathog 6: e1000959. 20585634 10.1371/journal.ppat.1000959 PMC2891831

[27] Klotman ME, Rapista A, Teleshova N, Micsenyi A, Jarvis GA, et al (2008) Neisseria gonorrhoeae-induced human defensins 5 and 6 increase HIV infectivity: role in enhanced transmission. J Immunol 180: 6176–6185. 18424739 10.4049/jimmunol.180.9.6176 PMC3042429

[28] Rapista A, Ding J, Benito B, Lo YT, Neiditch MB, et al (2011) Human defensins 5 and 6 enhance HIV-1 infectivity through promoting HIV attachment. Retrovirology 8: 45. 21672195 10.1186/1742-4690-8-45 PMC3146398

[29] Smith JG, Nemerow GR (2008) Mechanism of adenovirus neutralization by Human α-defensins. Cell Host Microbe 3: 11–19. 18191790 10.1016/j.chom.2007.12.001

[30] Wang W, Owen SM, Rudolph DL, Cole AM, Hong T, et al (2004) Activity of α- and θ-defensins against primary isolates of HIV-1. J Immunol 173: 515–520. 15210812 10.4049/jimmunol.173.1.515

[31] Yahi N, Fantini J, Baghdiguian S, Chermann JC (1991) Human T-lymphoblastoid cells selected for growth in serum-free medium provide new tools for study of HIV replication and cytopathogenicity. J Virol Methods 34: 193–207. 1725173 10.1016/0166-0934(91)90099-l

[32] O'Doherty U, Swiggard WJ, Malim MH (2000) Human immunodeficiency virus type 1 spinoculation enhances infection through virus binding. J Virol 74: 10074–10080. 11024136 10.1128/jvi.74.21.10074-10080.2000 PMC102046

[33] Mandel ID (1972) Mucosal secretions: chemico-physical analysis. In: C.V. Mosby Co. Saliva. 242–251.

[34] Porter EM, van Dam E, Valore EV, Ganz T (1997) Broad-spectrum antimicrobial activity of human intestinal defensin 5. Infect Immun 65: 2396–2401. 9169780 10.1128/iai.65.6.2396-2401.1997 PMC175332

[35] Berger EA, Murphy PM, Farber JM (1999) Chemokine receptors as HIV-1 coreceptors: roles in viral entry, tropism, and disease. Ann Rev Immunol 17: 657–700. 10358771 10.1146/annurev.immunol.17.1.657

[36] Lusso P, Cocchi F, Balotta C, Markham PD, Louie A, et al (1995) Growth of macrophage-tropic and primary human immunodeficiency virus type 1 (HIV-1) isolates in a unique CD4+ T-cell clone (PM1): failure to downregulate CD4 and to interfere with cell-line-tropic HIV-1. J Virol 69: 3712–3720. 7745720 10.1128/jvi.69.6.3712-3720.1995 PMC189087

[37] McKnight A, Wilkinson D, Simmons G, Talbot S, Picard L, et al (1997) Inhibition of human immunodeficiency virus fusion by a monoclonal antibody to a coreceptor (CXCR4) is both cell type and virus strain dependent. J Virol 71: 1692–1696. 8995702 10.1128/jvi.71.2.1692-1696.1997 PMC191233

[38] Brelot A, Heveker N, Adema K, Hosie MJ, Willett B, et al (1999) Effect of mutations in the second extracellular loop of CXCR4 on its utilization by human and feline immunodeficiency viruses. J Virol 73: 2576–2586. 10074102 10.1128/jvi.73.4.2576-2586.1999 PMC104012

[39] Baribaud F, Edwards TG, Sharron M, Brelot A, Heveker N, et al (2001) Antigenically distinct conformations of CXCR4. J Virol 75: 8957–8967. 11533159 10.1128/JVI.75.19.8957-8967.2001 PMC114464

[40] Lehrer RI (2007) Multispecific myeloid defensins. Curr Opin Hematol 14: 16–21. 17133095 10.1097/00062752-200701000-00005

[41] Hazrati E, Galen B, Lu W, Wang W, Ouyang Y, et al (2006) Human α- and β-defensins block multiple steps in herpes simplex virus infection. J Immunol 177: 8658–8666. 17142766 10.4049/jimmunol.177.12.8658

[42] Tanabe H, Ouellette AJ, Cocco MJ, Robinson WE Jr (2004) Differential effects on human immunodeficiency virus type 1 replication by α-defensins with comparable bactericidal activities. J Virol 78: 11622–11631. 15479803 10.1128/JVI.78.21.11622-11631.2004 PMC523300

[43] de Leeuw E, Burks SR, Li X, Kao JP, Lu W (2007) Structure-dependent functional properties of human defensin 5. FEBS Lett 581: 515–520. 17250830 10.1016/j.febslet.2006.12.036 PMC1832120

[44] Grigat J, Soruri A, Forssmann U, Riggert J, Zwirner J (2007) Chemoattraction of macrophages, T lymphocytes, and mast cells is evolutionarily conserved within the human α-defensin family. J Immunol 179: 3958–3965. 17785833 10.4049/jimmunol.179.6.3958

[45] Porter E, Yang H, Yavagal S, Preza GC, Murillo O, et al (2005) Distinct defensin profiles in Neisseria gonorrhoeae and Chlamydia trachomatis urethritis reveal novel epithelial cell-neutrophil interactions. Infect Immun 73: 4823–4833. 16040996 10.1128/IAI.73.8.4823-4833.2005 PMC1201278

[46] Davis HE, Rosinski M, Morgan JR, Yarmush ML (2004) Charged polymers modulate retrovirus transduction via membrane charge neutralization and virus aggregation. Biophys J 86: 1234–1242. 14747357 10.1016/S0006-3495(04)74197-1 PMC1303915

[47] Lehrer RI, Jung G, Ruchala P, Andre S, Gabius HJ, et al (2009) Multivalent binding of carbohydrates by the human α-defensin, HD5. J Immunol 183: 480–490. 19542459 10.4049/jimmunol.0900244

[48] Appay V, Rowland-Jones SL (2001) RANTES: a versatile and controversial chemokine. Trends Immunol 22: 83–87. 11286708 10.1016/s1471-4906(00)01812-3

[49] Jameson BA, Rao PE, Kong LI, Hahn BH, Shaw GM, et al (1988) Location and chemical synthesis of a binding site for HIV-1 on the CD4 protein. Science 240: 1335–1339. 2453925 10.1126/science.2453925

[50] Cardozo T, Kimura T, Philpott S, Weiser B, Burger H, et al (2007) Structural basis for coreceptor selectivity by the HIV type 1 V3 loop. AIDS Res Hum Retrov 23: 415–426. 10.1089/aid.2006.0130 17411375

[51] Cocchi F, DeVico AL, Garzino-Demo A, Cara A, Gallo RC, et al (1996) The V3 domain of the HIV-1 gp120 envelope glycoprotein is critical for chemokine-mediated blockade of infection. Nat Med 2: 1244–1247. 8898753 10.1038/nm1196-1244

[52] Quinones-Mateu ME, Lederman MM, Feng Z, Chakraborty B, Weber J, et al (2003) Human epithelial β-defensins 2 and 3 inhibit HIV-1 replication. AIDS 17: F39–48. 14571200 10.1097/00002030-200311070-00001

[53] Feng Z, Dubyak GR, Lederman MM, Weinberg A (2006) Cutting edge: human β defensin 3–a novel antagonist of the HIV-1 coreceptor CXCR4. J Immunol 177: 782–786. 16818731 10.4049/jimmunol.177.2.782

[54] Zhou N, Fang J, Mukhtar M, Acheampong E, Pomerantz RJ (2004) Inhibition of HIV-1 fusion with small interfering RNAs targeting the chemokine coreceptor CXCR4. Gene Ther 11: 1703–1712. 15306840 10.1038/sj.gt.3302339

[55] Anderson J, Akkina R (2005) HIV-1 resistance conferred by siRNA cosuppression of CXCR4 and CCR5 coreceptors by a bispecific lentiviral vector. AIDS Res Ther 2: 1. 15813990 10.1186/1742-6405-2-1 PMC1074340

[56] Ghosh D, Porter E, Shen B, Lee SK, Wilk D, et al (2002) Paneth cell trypsin is the processing enzyme for human defensin-5. Nat Immunol 3: 583–590. 12021776 10.1038/ni797

[57] Chen L, Kwon YD, Zhou T, Wu X, O'Dell S, et al (2009) Structural basis of immune evasion at the site of CD4 attachment on HIV-1 gp120. Science 326: 1123–1127. 19965434 10.1126/science.1175868 PMC2862588

[58] Chang TL, Vargas J Jr, DelPortillo A, Klotman ME (2005) Dual role of α-defensin-1 in anti-HIV-1 innate immunity. J Clin Invest 115: 765–773. 15719067 10.1172/JCI200521948 PMC548697

[59] Brenchley JM, Douek DC (2008) HIV infection and the gastrointestinal immune system. Mucosal Immunol 1: 23–30. 19079157 10.1038/mi.2007.1 PMC2777614

[60] Zaragoza MM, Sankaran-Walters S, Canfield DR, Hung JK, Martinez E, et al (2011) Persistence of gut mucosal innate immune defenses by enteric α-defensin expression in the simian immunodeficiency virus model of AIDS. J Immunol 186: 1589–1597. 21178012 10.4049/jimmunol.1002021 PMC4052980

[61] Lagenaur LA, Sanders-Beer BE, Brichacek B, Pal R, Liu X, et al (2011) Prevention of vaginal SHIV transmission in macaques by a live recombinant Lactobacillus. Mucosal Immunol 4: 648–657. 21734653 10.1038/mi.2011.30 PMC3433722

[62] Kaul R, Rebbapragada A, Hirbod T, Wachihi C, Ball TB, et al (2008) Genital levels of soluble immune factors with anti-HIV activity may correlate with increased HIV susceptibility. AIDS 22: 2049–2051. 18784472 10.1097/QAD.0b013e328311ac65 PMC2650776

[63] Iqbal SM, Kaul R (2008) Mucosal innate immunity as a determinant of HIV susceptibility. Am J Reprod Immunol 59: 44–54. 18154595 10.1111/j.1600-0897.2007.00563.x

[64] Eade CR, Wood MP, Cole AM (2012) Mechanisms and modifications of naturally occurring host defense peptides for anti-HIV microbicide development. Current HIV research 10: 61–72. 22264047 10.2174/157016212799304580 PMC4270272

[65] Lusso P, Earl PL, Sironi F, Santoro F, Ripamonti C, et al (2005) Cryptic nature of a conserved, CD4-inducible V3 loop neutralization epitope in the native envelope glycoprotein oligomer of CCR5-restricted, but not CXCR4-using, primary human immunodeficiency virus type 1 strains. J Virol 79: 6957–6968. 15890935 10.1128/JVI.79.11.6957-6968.2005 PMC1112133

[66] Scarlatti G, Tresoldi E, Bjorndal A, Fredriksson R, Colognesi C, et al (1997) In vivo evolution of HIV-1 co-receptor usage and sensitivity to chemokine-mediated suppression. Nat Med 3: 1259–1265. 9359702 10.1038/nm1197-1259

[67] Zhou Y, Zhang H, Siliciano JD, Siliciano RF (2005) Kinetics of human immunodeficiency virus type 1 decay following entry into resting CD4+ T cells. J Virol 79: 2199–2210. 15681422 10.1128/JVI.79.4.2199-2210.2005 PMC546571

[68] Moore JP, McKeating JA, Weiss RA, Sattentau QJ (1990) Dissociation of gp120 from HIV-1 virions induced by soluble CD4. Science 250: 1139–1142. 2251501 10.1126/science.2251501

[69] Nussbaum O, Broder CC, Berger EA (1994) Fusogenic mechanisms of enveloped-virus glycoproteins analyzed by a novel recombinant vaccinia virus-based assay quantitating cell fusion- dependent reporter gene activation. J Virol 68: 5411–5422. 8057423 10.1128/jvi.68.9.5411-5422.1994 PMC236941

